# Supplementary material for: Informative and adaptive distances and summary statistics in sequential approximate Bayesian computation
Source: PLoS One. 2023 May 22;18(5):e0285836. doi: 10.1371/journal.pone.0285836 (PMC10202307; doi:10.1371/journal.pone.0285836)
Supplement: S1 File — (PDF) [file pone.0285836.s001.pdf]

Supplementary information: Informative and adaptive distances and summary statistics in sequential approximate Bayesian computation

Yannik Schälte [1,2,3] and Jan Hasenauer [1,2,3,*]

[1] Institute of Computational Biology, Helmholtz Zentrum München, 85764 Neuherberg, Germany

[2] Center for Mathematics, Technische Universität München, 85748 Garching, Germany

[3] Faculty of Mathematics and Natural Sciences, Rheinische Friedrich-Wilhelms-Universität Bonn, 53115 Bonn, Germany

[*] To whom correspondence should be addressed (jan.hasenauer@uni-bonn.de)

1 Optimal summary statistics to recover distribution features

Theorem 1. *Denote the joint distribution of parameters and data* $\Theta, Y \sim \pi(\theta, y)$, *with prior marginal* $\pi(\theta) = \int \pi(\theta, y)\, dy$, *likelihood* $\pi(y|\theta) = \pi(\theta, y)/\pi(\theta)$, *and posterior* $\pi(\theta|y) = \pi(\theta, y)/\pi(y) = \pi(y|\theta)\pi(\theta)/\pi(y)$. *Given a parameter transformation* $\lambda : \mathbb{R}^{n_\theta} \to \mathbb{R}^{n_\lambda}$ *such that* $\mathbb{E}_{\pi(\theta)}[|\lambda(\theta)|] < \infty$, *define summary statistics as the conditional expectation*

$$s(y) := \mathbb{E}[\lambda(\Theta)|Y = y] = \int \lambda(\theta)\pi(\theta|y)\, d\theta\, .$$

Given observed data y_{obs}, *acceptance threshold* ε, *and assuming the distance metric* $d(s(y), s(y_{obs})) = \|s(y) - s(y_{obs})\|$ *is norm-induced, denote the ABC posterior distribution*

$$\pi_{\mathrm{ABC},\varepsilon}(\theta|s(y_{obs})) \propto \int I[\|s(y) - s(y_{obs})\| \leq \varepsilon]\pi(y|\theta)\, dy \cdot \pi(\theta).$$

Then, it holds

$$\left\|\mathbb{E}_{\pi_{\mathrm{ABC},\varepsilon}}[\lambda(\Theta)|s(y_{obs})] - s(y_{obs})\right\| \leq \varepsilon, \tag{1}$$

and therefore

$$\lim_{\varepsilon\to 0} \mathbb{E}_{\pi_{\mathrm{ABC},\varepsilon}}[\lambda(\Theta)|s(y_{obs})] = \mathbb{E}[\lambda(\Theta)|Y = y_{obs}]. \tag{2}$$

Proof. Based on Fearnhead and Prangle [2012] and Jiang et al. [2017], a simple extension of the argumentation in the latter. Note that $s(y)$ is almost surely finite due to $\mathbb{E}[|\lambda(\theta)|] < \infty$ and Fubini's Theorem. As for the induced σ-algebras holds $\sigma(s(Y)) \subset \sigma(Y)$, $s(Y)$ is also a version of the conditional expectation $\mathbb{E}[\lambda(\Theta)|s(Y)]$, since

$$s(Y) = \mathbb{E}[s(Y)|s(Y)] = \mathbb{E}[\mathbb{E}[\lambda(\Theta)|Y]|s(Y)] = \mathbb{E}[\lambda(\Theta)|s(Y)]$$

by, respectively, measurability, definition, and tower property. Thus, denoting the acceptance region

$$A = \{\|s(Y) - s(y_{\text{obs}})\| \leq \varepsilon\} \in \sigma(s(Y)),$$

with $\mathbb{E}[\lambda(\Theta)|A] = \mathbb{E}[\lambda(\Theta)\mathbb{1}_A]/\mathbb{E}[\mathbb{1}_A]$, we have

$$\mathbb{E}_{\text{ABC},\varepsilon}[\lambda(\Theta)|s(y_{\text{obs}})] = \mathbb{E}[\lambda(\Theta)|A] = \mathbb{E}[s(Y)|A],$$

such that by Jensen's inequality, given convexity of the norm,

$$\left\|\mathbb{E}_{\pi_{\text{ABC},\varepsilon}}[\lambda(\Theta)|y_{\text{obs}}] - s(y_{\text{obs}})\right\| = \|\mathbb{E}[s(Y) - s(y_{\text{obs}})|A]\| \leq \mathbb{E}[\|s(Y) - s(y_{\text{obs}})\|\,|A] \leq \varepsilon.$$

(2) then follows directly from (1) by definition of $s(y_{\text{obs}})$. □

Therefore, e.g. for $\lambda(\theta) = (\theta^1, \ldots, \theta^k)$, the corresponding first k moments of the true posterior distribution are recovered by an ABC analysis employing the posterior expectation $s(y) = \mathbb{E}[\lambda(\Theta)|Y = y]$ as summary statistic, for $\varepsilon \to 0$. For $k \to \infty$, $\varepsilon \to 0$, and e.g. assuming existence of moment-generating functions, the approximate posterior converges to the true posterior.

2 Effective sample sizes

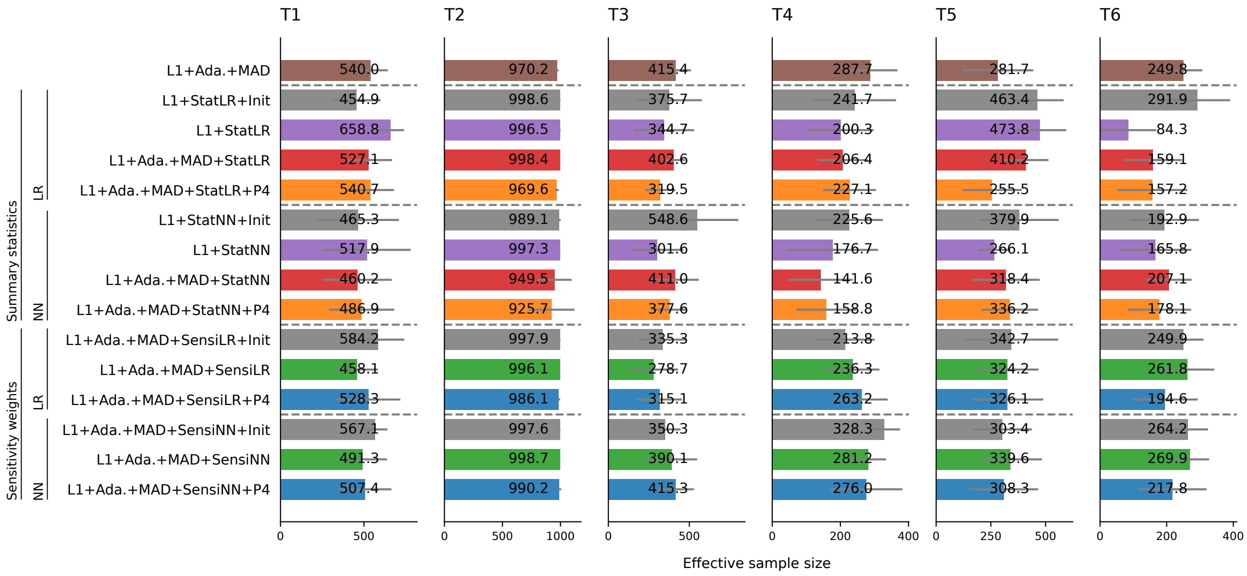

Figure S1: Effective sample sizes (ESS) for models T1-6. Given particles $P_{n_t} = \{(\theta_i, w_i)\}_i$ accepted in the last generation, the ESS is defined as $\text{ESS} = (\sum_i w_i)^2 / \sum_i w_i^2$ [Martino et al., 2017]. Shown are means and standard deviations (grey error bars) over all performed runs.

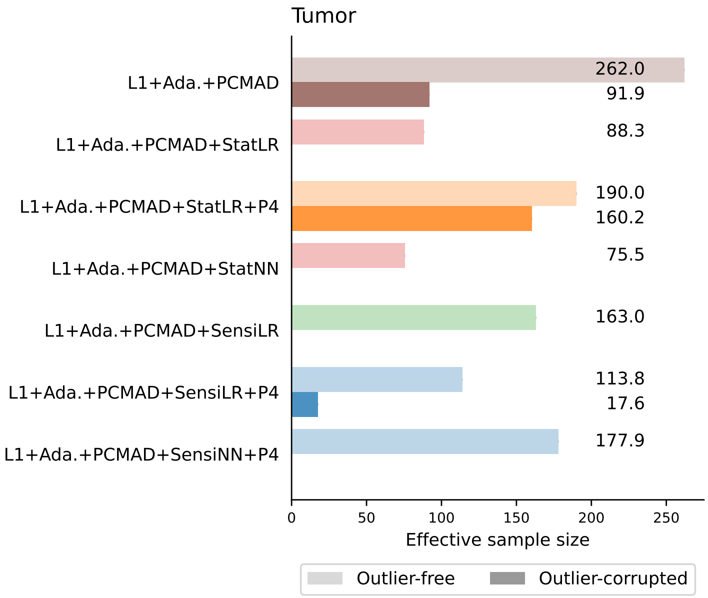

Figure S2: Effective sample sizes (ESS) for the tumor model, on outlier-free (light bars) and outlier-corrupted (dark bars) data, for selected settings. Note that on outlier-corrupted data, only three settings were run.

3 Differentiability of the data-parameter regression model mapping

One core assumption of the sensitivity weighting (Main Manuscript, Section 2.2.2) is that the employed regression model is (sub-)differentiable, such that gradients can be robustly calculated.

To verify this, we consider in this section first the demonstration example, and second a modified test problem T4. The demonstration example consists of different normally distributed observables with means related linearly or non-linearly to different parameters. T4 is a discrete Markov-jump process model of predator-prey dynamics. Here, we modified the original model to have zero noise, leaving integer-valued observables.

For each problem, we created a training set of $10,000$ tuples of parameters and simulated data (θ, y), sampled i.i.d. from the prior distribution. We fitted first a linear regression model, and second a neural network model with four hidden layers of dimension 100, to the mapping $y \mapsto \theta$.

In Figures S3 and S4, we plotted the predictions of the two regression models, varying each input one at a time. It can be observed that the outputs vary continuously as piecewise differentiable functions of their inputs. In the case of the second problem, they thus interpolate smoothly between the integer-valued inputs.

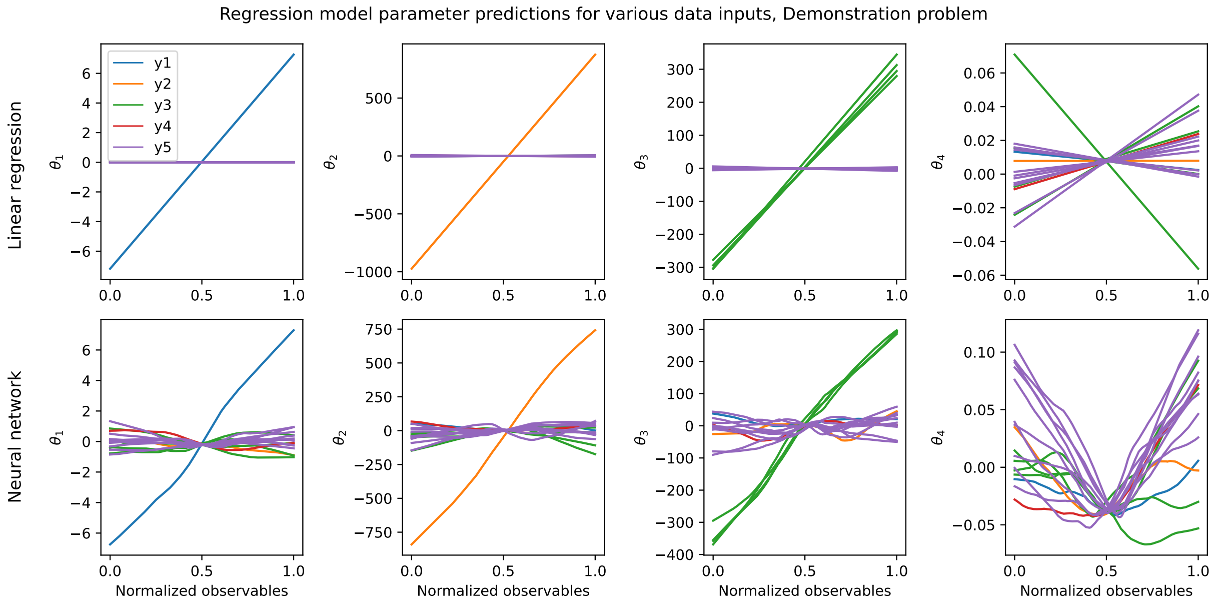

Figure S3: Regression model parameter predictions for various data inputs, Demonstration problem. Base input vector were a given set of observed data. Each line denotes the prediction values obtained by varying a given input (observable coordinate) one at a time, over a range of values informed by the prior range, normalized here to a common range $[0, 1]$. From left to right, predictions for different parameters are shown. Top: Linear regression model. Bottom: Neural network model with 4 hidden layers of dimension 100. The regression model were trained on $10,000$ prior samples.
Besides continuity of the predictions, one can in particular observe how $\theta_1, \theta_2, \theta_3$ show, respectively, a clear dependence on y_1, y_2, y_3, while a functional description $y \mapsto \theta_4$ cannot be found (as this requires the consideration of higher-order moments). Yet, for more complex non-linear problems, analyses as shown here should not be overinterpreted in terms of predictions, as we consider a relatively small training set with simple regression models on the full prior range.

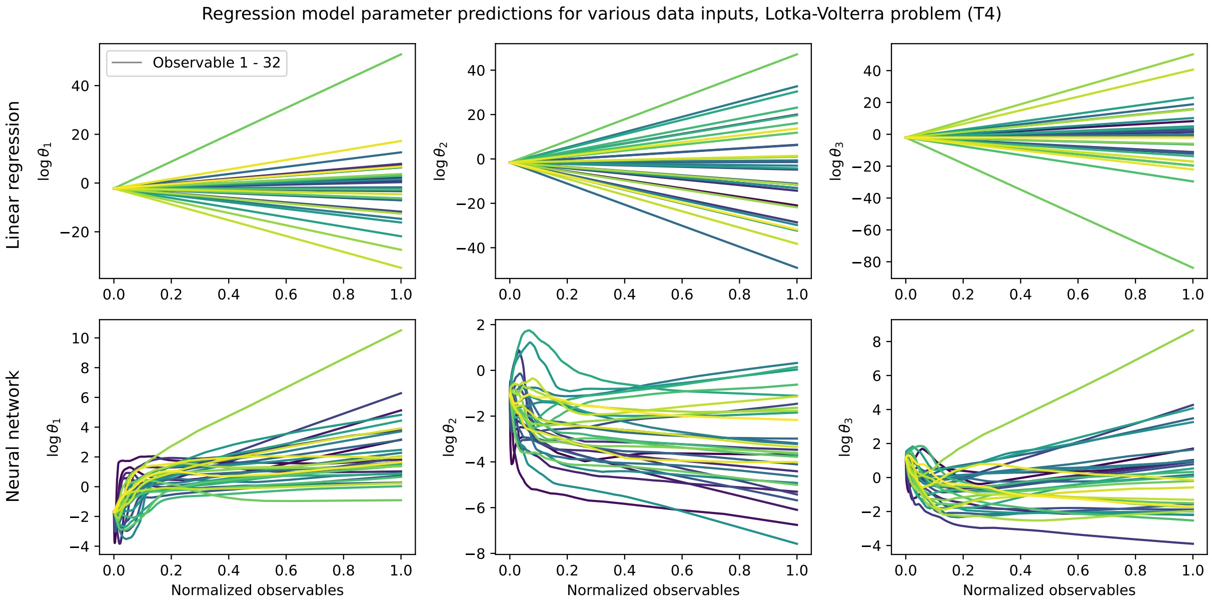

Figure S4: Similar to Figure S3, for the Lotka-Volterra problem T4.

References

Fearnhead, P. and Prangle, D. Constructing summary statistics for approximate Bayesian computation: semi-automatic approximate Bayesian computation. *J. R. Stat. Soc. B*, 74(3):419–474, 2012.

Jiang, B. et al. Learning summary statistic for approximate Bayesian computation via deep neural network. *Statistica Sinica*, pages 1595–1618, 2017.

Martino, L. et al. Effective sample size for importance sampling based on discrepancy measures. *Signal Processing*, 131:386–401, 2017.
