# Supplementary material for: Informative and adaptive distances and summary statistics in sequential approximate Bayesian computation
Source: PLoS One. 2023 May 22;18(5):e0285836. doi: 10.1371/journal.pone.0285836 (PMC10202307; doi:10.1371/journal.pone.0285836)
Supplement: Attachment — Submitted filename: JPONE_D-22-20368_report.pdf [file pone.0285836.s002.pdf]

# PONE-D-22-20368

September 27, 2022

## 1 Summary

The authors proposes the use of sensitivity weights obtained from regression adjustment within ABC to deduce the presence of identification issues and define an adaptive distance for ABC-based inferences. The authors present their approach using a mix of toy examples, and more realistic examples. The papers is interesting and potentially publishable, at least after the material is appropriately revised.

## 2 General Comments

**(1) Identifiable Issues.** The authors help motivate their approach by stating that "an inverse mapping may not exist, e.g. when parameters are not globally identified," and that the sensitivity weights proposed can help identify this issue. A similar point is also made in the conclusion, where they say that they "introduced augmented regression targets to overcome parameter non-identifiablity".

1.1) I have seen identification issues occur in ABC for two main reasons: the summaries and model combination are not sufficiently rich to allow ABC to identify the parameters; their exists no parameter value under which the summaries can match the observed data. From the statement in the introduction, at this stage in the paper it is not clear which of the two scenarios the authors intend this statement to identity.

1.2) The reader who is familiar with ABC methods would appreciate some discussion on which of the above two scenarios the authors are talking about. In the case of the former, identification can often be salvaged by adding (appropriate) extra information (Li and Fearnhead, 2018, and Frazier et al., 2018). In the latter case, there is generally no way to salvage identification and regression adjustment is known to produce unreliable results; Frazier et al. (2020), Schmon et al. (2020).

**(2)** I found the authors switching between the presentation of ABC based on summaries and full data distances in sections 2.1.1.-2.1.3 a bit confusing. I understand the use of this in Section 2.1.2-2.1.3, but I don't really see the reason for presenting this in terms of the "data" and not the summaries. In any meaningful application we must rely on summaries, so surely these algorithms should be presented in a common way?

**(3)**It is unclear to me how/if the authors approach to calculating sensitivities will be useful when the summaries are discontinuous as a function of the parameters. The use of central finite

differences will not help here. The authors, however, make no mention of this case even though in the g-and-k example quantile statistics, which are discontinuous as a function of the parameters, are commonly used in this model, and thus derivatives of such summaries cannot be consistently estimated via finite-differences, at least not in an unbiased manner. Some discussion on this would be appreciated; e.g., what do the authors suggest to do when the summaries used are not differentiable?

**(5)** I have several comments about section 2.2.3.

- The nature of the inverse mapping you discuss is not the notion of the inverse mapping commonly used in ABC; see, e.g., Li and Fearnhead (2018) and Frazier et al. (2018), which is a mapping from the model to the summaries. I found this confusing, and other readers may as well so please note this distinction in the text.
- What you are actually discussing seems to be that the model you have assumed is itself not identified: the prior assumption is such that you can only identify $\pm\theta$. This should be made clearer so as not to confuse the reader.
- Unless I have missed something, the authors solution of expanding the set of basis functions for the regression summaries beyond the first order is precisely the suggestion given in Fearnhead and Prangle (2012). Why is this not acknowledged? In particular, the authors suggest to take higher-order moments, and the precise idea discussed in FP is to approximate the posterior expectation with the set $\eta = (\eta, \eta^2, \eta^3, \eta^4)^\top$, see page 428 in Fearnhead and Prangle (2012), and page 196 in Nunes and Prangle (2015).
- It's also important to note that the idea of expanding the basis with powers will not solve all issues. For instance, this approach can exacerbate the identification issue when the model is misspecified, as the larger number of summaries itself exacerbates the presence of model misspecification. As such, I would hazard a guess that this approach would perform poorly when the model is misspecified. See Frazier et al. (2020) and Schmon et al. (2020) for details and examples.

**Minor Comments**

1. I do not see where the notation $S_{i_y,i_\theta}$ has been defined. While it is fairly obvious what it is, it would be better to have it defined properly.
2. The plot in Figure 4 is difficult to discern given the scale. Please correct this.

# References


[1] Li, Wentao, and Paul Fearnhead. "On the asymptotic efficiency of approximate Bayesian computation estimators." Biometrika 105, no. 2 (2018): 285-299.

[2] Frazier, David T., Gael M. Martin, Christian P. Robert, and Judith Rousseau. "Asymptotic properties of approximate Bayesian computation." Biometrika 105, no. 3 (2018): 593-607.

[3] Frazier, David T., Christian P. Robert, and Judith Rousseau. ”Model misspecification in approximate Bayesian computation: consequences and diagnostics.” Journal of the Royal Statistical Society: Series B (Statistical Methodology) 82, no. 2 (2020): 421-444.

[4] Fearnhead, Paul, and Dennis Prangle. ”Constructing summary statistics for approximate Bayesian computation: semi-automatic approximate Bayesian computation.” Journal of the Royal Statistical Society: Series B (Statistical Methodology) 74, no. 3 (2012): 419-474.

[5] Nunes, Matthew Alan, and Dennis Prangle. ”abctools: an R package for tuning approximate Bayesian computation analyses.” The R Journal 7, no. 2 (2015): 189-205.

[6] Schmon, Sebastian M., Patrick W. Cannon, and Jeremias Knoblauch. ”Generalized posteriors in approximate bayesian computation.” arXiv preprint arXiv:2011.08644 (2020).
