# Supplementary material for: Informative and adaptive distances and summary statistics in sequential approximate Bayesian computation
Source: PLoS One. 2023 May 22;18(5):e0285836. doi: 10.1371/journal.pone.0285836 (PMC10202307; doi:10.1371/journal.pone.0285836)
Supplement: Attachment — Submitted filename: review.pdf [file pone.0285836.s003.pdf]

Review The authors introduce regression-based summary statistics to overcome three problems that may arise in ABC:

1. From partly uninformative data for scale-normalized distances
2. From heterogeneous parameter scales for regression-based summary statistics
3. From parameter non-identifiability for regression model adequacy

The manuscript contributions are partly based on NN models. This is an important application which has received broad attention recently [1]. The manuscript's findings are of interest to the readers of PLOS ONE, and I recommend proceeding with the manuscript publication after a few issues listed below are addressed.

Model selection Does Theorem 1 in the supplementary information imply conditions for model selection, e.g., in the sense of Marin *et al.* [3]? You mention model selection as part of the future work in the discussion of the manuscript. Model selection would make *pyABC* powerful.

Curse of dimensionality The results in the manuscript are applied to a somewhat large number of low-dimensional examples. I miss some candid insight on where the limitations are while using the manuscript results in high dimensional parameter space. How would the NN model need to be adapted? ("*...NN models were considered with a single hidden layer of dimension*$[(n_y + n_\theta)/2]$, *with ReLU activation function, using ADAM stochastic gradient descent for optimization, and early stopping to avoid overfitting, with a 10% validation set....*).

Non-identifiability I fail to understand the non-identifiability remedy presented in the results. Wouldn't it make sense to say a model is identifiable is the parameter to observable mapping (in the zero noise limit) is

injective, e.g., in the sense of Bellu *et al.* [2], and have a separate name for the result shown in the manuscript? If we a have population growth model with both intrinsic growth rate and carrying capacity, but the corresponding data only informs the growth regime, then the carrying capacity is non-identifiable. Is it possible to apply the manuscript findings to make the carrying capacity identifiable?

References

[1] M. A. Beaumont. Approximate bayesian computation. *Annual review of statistics and its application*, 6:379–403, 2019.

[2] G. Bellu, M. P. Saccomani, S. Audoly, and L. D'Angiò. Daisy: A new software tool to test global identifiability of biological and physiological systems. *Computer methods and programs in biomedicine*, 88(1):52–61, 2007.

[3] J.-M. Marin, N. S. Pillai, C. P. Robert, and J. Rousseau. Relevant statistics for bayesian model choice. *Journal of the Royal Statistical Society: Series B (Statistical Methodology)*, 76(5):833–859, 2014.
