# Supplementary material for: Informative and adaptive distances and summary statistics in sequential approximate Bayesian computation
Source: PLoS One. 2023 May 22;18(5):e0285836. doi: 10.1371/journal.pone.0285836 (PMC10202307; doi:10.1371/journal.pone.0285836)
Supplement: Attachment — Submitted filename: Response_Informative.pdf [file pone.0285836.s004.pdf]

Author's response to editor's and reviewers' comments

For the paper: "*Informative and adaptive distances and summary statistics in sequential approximate Bayesian computation*".

We thank the editor and the reviewers for their comments. Based on their remarks, we have revised the manuscript and the supplementary information. We addressed all their comments and resolved the raised issues. Our point-by-point response is provided below.

The teal boxes contain the editor's and the reviewers' comments, while the yellow boxes contain the changes made in the manuscript. To provide some context, changes (blue) are sometimes accompanied by unchanged text (black).

Response to Reviewer 1

Summary

The authors proposes the use of sensitivity weights obtained from regression adjustment within ABC to deduce the presence of identification issues and define an adaptive distance for ABC-based inferences. The authors present their approach using a mix of toy examples, and more realistic examples. The papers is interesting and potentially publishable, at least after the material is appropriately revised.

We thank the reviewer for this assessment.

General comments

(1) Identifiable Issues. The authors help motivate their approach by stating that "an inverse mapping may not exist, e.g. when parameters are not globally identified," and that the sensitivity weights proposed can help identify this issue. A similar point is also made in the conclusion, where they say that they "introduced augmented regression targets to overcome parameter non- identifiablity".
(1.1) I have seen identification issues occur in ABC for two main reasons: the summaries and model combination are not sufficiently rich to allow ABC to identify the parameters; their exists no parameter value under which the summaries can match the observed data. From the statement in the introduction, at this stage in the paper it is not clear which of the two scenarios the authors intend this statement to identity.

We thank the reviewer for pointing out the missing clarity. What we mean here is identifiability of parameters given data, i.e. whether dissimilar parameters can generate similar data, thus the former above-described scenario. Yet, it might not only be an issue of the used summary statistics but also of the original data themselves. It is even possible that some parameters are structurally non-identifiable from the observables, i.e. no resolution or quality of data from given model outputs can allow to structurally identify the parameters [Wieland et al., 2021]. We show in the paper that in this case regression-based approaches targeting only the posterior mean fail to extract valuable information, while approaches aiming at a better approximation of the posterior overcome this issue.

We have clarified the description as follows:

Changes in the manuscript:

Initial submission: A further problem with regression-based statistics is that an inverse mapping may not always exist, e.g. when parameters are not globally identifiable.

Revised submission: A further problem with regression-based statistics is that a unique inverse mapping from data to parameters may not always exist even in the noise-free limit, resulting e.g. in a multi-modal posterior distribution, or plateaus of combinations of parameter values achieving the same posterior value. This is in particular the case when the observable model outputs and data are not rich enough to allow to structurally identify the parameters globally [Wieland et al., 2021].

(1.2) The reader who is familiar with ABC methods would appreciate some discussion on which of the above two scenarios the authors are talking about. In the case of the former, identification can often be salvaged by adding (appropriate) extra information (Li and Fearnhead, 2018, and Frazier et al., 2018). In the latter case, there is generally no way to salvage identification and regression adjustment is known to produce unreliable results; Frazier et al. (2020), Schmon et al. (2020).

We thank the reviewer for this comment, which we believe should indeed be discussed in the text. An example of global structural non-identifiability is a model $\theta \mapsto y := \theta^2$, which is also discussed as the first example in the manuscript. The convergence results given in Li and Fearnhead [2018] and Frazier et al. [2018] assume identifiability, which here does not hold. Assuming such a structural global non-identifiability, there is simply no further information available that would allow to break the symmetry. In biochemical systems, non-identifiability occurs frequently when only a subset or combinations of species are measurable – in this case there is simply no way of fixing the identifiability problem beyond fixing single parameters and reparameterization. see e.g. Fröhlich et al. [2018] for an example.

We have clarified the description in the Methods Section 2.2.3 as follows:

Changes in the manuscript:

Initial submission: A problem with inverse regression models of parameters on data is that such a mapping may not exist. For example, consider a quadratic model $y \sim \mathcal{N}(\theta^2, 0.1^2)$, with prior $\theta \sim U[-1, 1]$, and observed data $y_{\text{obs}} = 0.7$. [...]
A solution is to consider transformations $\lambda(\theta)$ of the parameters [...]

Revised submission: A problem with inverse regression models of parameters on data is that such a mapping may not exist. For example, consider a quadratic model $y \sim \mathcal{N}(\theta^2, 0.1^2)$, with prior $\theta \sim U[-1, 1]$, and observed data $y_{\text{obs}} = 0.7$. Given the data, the underlying parameter cannot be uniquely identified. [...]
In general, non-identifiability can be tackled in various ways, e.g. by fixing some parameters to constant values, reparameterization, or by generating additional data to break the symmetry [Wieland et al., 2021]. However, this requires an in-depth and iterative model analysis. Even for deterministic models based on ordinary differential equation, this is already a challenging task [Villaverde et al., 2021], while ABC is commonly applied to highly complex stochastic models. Further, one ends up with an altered inference problem, which renders the analysis of prediction uncertainties more involved [Villaverde et al., 2021].
Here we propose an approach that is able to work with the original, unaltered, problem formulation, including potential non-identifiabilities. We solve the problem by considering transformations $\lambda(\theta)$ of the parameters. [...]

We consider the prior as part of the problem and not something to update based on the model

or observed data. In this context, see also our discussion in the manuscript, e.g. in l. 149:

In Jiang et al. [2017], step 1 is omitted, using the prior directly, in one case constrained to an identifiable region.

and in l. 812

While Fearnhead and Prangle [2012] update the prior to a typical range of values observed in the pilot run, we consider the prior as part of the problem formulation, and thus do not update it. In generations $t \geq t_{train}$ the proposal distributions g_t will usually anyway mostly suggest values within the training domain range.

Further, we have updated, also in response to another review comment, our discussion of the identifiability issue in the discussion as follows:

Changes in the manuscript:

Initial submission: -

Revised submission: While augmenting regression targets was essential to render inverse regression model based approaches applicable to non-identifiable forward models, one could further study the ramifications of the increased summary statistics dimension, as well as methods to automatically adjust the set of applied transformations. In particular, for multivariate problems it may be necessary to also consider combinations of parameters to render the inverse problem identifiable. While we showed that our approach also performed robustly in the presence of outliers for a given model, its applicability in general under model misspecification could be studied further [Frazier et al., 2020, Schmon et al., 2020].

(2) I found the authors switching between the presentation of ABC based on summaries and full data distances in sections 2.1.1.-2.1.3 a bit confusing. I understand the use of this in Section 2.1.2-2.1.3, but I don't really see the reason for presenting this in terms of the "data" and not the summaries. In any meaningful application we must rely on summaries, so surely these algorithms should be presented in a common way.

We thank the reviewer for this comment. A remark we had included must have gotten lost in a revision of the document. We have added the following clarification to Section 2.1.1:

Changes in the manuscript:

Initial submission: -

Revised submission: In this work, we will denote by y either the raw data, or assume it to already incorporate a mapping to (manually crafted) summary statistics. We will assess y in ABC directly via the use of weighted distance metrics, or on top of y automatically derive regression-based summary statistics s.

(3)It is unclear to me how/if the authors approach to calculating sensitivities will be useful when the summaries are discontinuous as a function of the parameters. The use of central finite differences will not help here. The authors, however, make no mention of this case even though in the g-and-k example quantile statistics, which are discontinuous as a function of the parameters, are commonly used in this model, and thus derivatives of such summaries cannot be consistently estimated via finite-differences, at least not in an unbiased manner. Some discussion on this would be appreciated; e.g., what do the authors suggest to do when the summaries used are not differentiable?

We thank the reviewer for this important comment. A core assumption of our approach is the differentiability of the employed regression model. However, in the first place we only require differentiability of the regression model, not of the modeled underlying quantities. For the considered linear and neural network based formulations, (sub-)differentiability is given. Yet, if the modeled underlying quantities are not differentiable, the question may arise how good an approximation the regression model can be. Gradients of a smooth approximation may still capture important relationships, however especially for discrete models, this may not always be the case.

For the g-and-k example, we are not sure whether we understood the reviewer's comment correctly. The distribution quantiles, used as statistics in our examples, are at least continuous in expectation as a function of the parameters, see Figure 1.

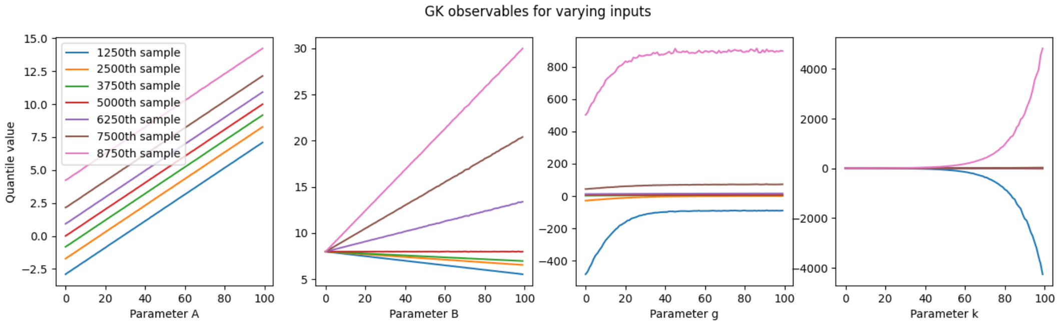

Figure 1: GK observables for varying inputs. The shown values are averages across 100 realizations (approximating $\mathbb{E}[y|\theta]$) for varying parameters θ. For each of the four parameters, its value was varied over its prior range $[0, 10]$, with the other parameters fixed to a random sample from the prior domain.

To study the aspect of discreteness, we considered a Markov-jump process model of Lotka-Volterra predator prey dynamics, which exhibits discrete integer-valued observables (on top of which we sampled Gaussian noise). In this case, we thus feed discrete inputs into the regression model. While it would be conceptually possible for the neural network model to learn a stepwise function in the inputs, we observed no such behavior in practice; rather, the neural network model smoothly interpolated between inputs (which of course also applied to the linear regression model).

To properly discuss this point, we added the following paragraph to discuss the method, and added a section to the Supplementary Information:

Changes in the manuscript:

Initial submission: -

Revised submission: It should be noted that in calculating sensitivity weights we make the assumption that the employed regression model is (sub-)differentiable, which clearly holds for the regression models employed in this work (see also the Supplementary Information, Section 3). Further, we implicitly assume that the employed smooth regression model provides an accurate approximation of the underlying expectations it aims to describe. In practice, it should be carefully evaluated whether such an approximation appears valid for the given forward model.

(5) I have several comments about section 2.2.3.

- The nature of the inverse mapping you discuss is not the notion of the inverse mapping commonly used in ABC; see, e.g., Li and Fearnhead (2018) and Frazier et al. (2018), which is a mapping from the model to the summaries. I found this confusing, and other readers may as well so please note this distinction in the text.

We are not entirely sure whether we understood this comment; in the referenced papers we cannot find a mention of "inverse mappings" or similar. We would be thankful for a clarification. To clarify what we mean by "inverse mapping", we rephrased our first mention of the notion (this change is also already contained in our response to a previous comment). We hope that this resolves the issue.

Changes in the manuscript:

Initial submission: A further problem with regression-based statistics is that an inverse mapping may not always exist.

Revised submission: A further problem with regression-based statistics is that a unique inverse mapping from data to parameters may not always exist even in the noise-free limit, resulting e.g. in a multi-modal posterior distribution, or plateaus of combinations of parameter values achieving the same posterior value. This is in particular the case when the observable model outputs and data are not rich enough to allow to structurally identify the parameters globally [Wieland et al., 2021].

- What you are actually discussing seems to be that the model you have assumed is itself not identified: the prior assumption is such that you can only identify $\pm\theta$. This should be made clearer so as not to confuse the reader.

Agreed, this is what we meant – the model parameters are not globally identifiable. We see that we should have been clearer on this and hope to have clarified this point in our responses above.

- Unless I have missed something, the authors solution of expanding the set of basis functions for the regression summaries beyond the first order is precisely the suggestion given in Fearnhead and Prangle (2012). Why is this not acknowledged? In particular, the authors suggest to take higher-order moments, and the precise idea discussed in FP is to approximate the posterior expectation with the set $\eta = (\eta, \eta^2, \eta^3, \eta^4)^T$, see page 428 in Fearnhead and Prangle (2012), and page 196 in Nunes and Prangle (2015).

In our understanding there is a major difference between the two considerations of moments in our manuscript and Fearnhead and Prangle [2012]: Fearnhead and Prangle [2012] augment the data y used as inputs to the regression model (i.e. as summary statistics) by e.g. higher-order moments $y \mapsto (y^1, \dots, y^4)$. This is because these may serve as additional regressors better describing the parameters via a linear relationship. This was no longer necessary in follow-up papers using regression models that can cover non-linear relationships. Yet, this cannot provide a solution in the situation that parameters cannot be described as functions of the data (or functions thereof). In contrast, what we do is not to augment the data, but to augment the parameters, e.g. $\theta \mapsto (\theta^1, \dots, \theta^4)$. As our quadratic example shows, this overcomes the

identifiability problem, as e.g. θ_4^2 can be described as a (linear) function of the data, whereas θ_4 cannot. This has the consequence that our summary statistics, the outputs of our regression model, made use of y_4 (i.e. the data corresponding to θ_4), while the approach by Fearnhead and Prangle [2012] mostly ignored these in its summary statistics, and thus learned little about θ_4.

We hope to have clarified this important distinction in the manuscript in Section 2.2.3:

Changes in the manuscript:

Initial submission: -

Revised submission: We would like to remark that here we augment the regression targets θ, while e.g. Blum et al. [2013], Fearnhead and Prangle [2012] instead augment the regressors y, e.g. by higher-order moments $y \mapsto (y^1, \ldots, y^4)$. Their approach allows to employ simple regression models, such as linear ones, on an increased feature space, which can be guided by model analysis and prior experimentation. However, it can conceptually not solve the problem tackled by our approach, that some parameters cannot be described as functions of the data (or transformations thereof). Of course, it would also be possible to augment regressors and regression targets simultaneously.

- It's also important to note that the idea of expanding the basis with powers will not solve all issues. For instance, this approach can exacerbate the identification issue when the model is misspecified, as the larger number of summaries itself exacerbates the presence of model misspecification. As such, I would hazard a guess that this approach would perform poorly when the model is misspecified. See Frazier et al. (2020) and Schmon et al. (2020) for details and examples.

We absolutely agree that the approaches we present in this manuscript, in particular the basis expansion, do not solve all issues. Model misspecification is certainly a complex topic on its own. How our approach performs under model misspecification is an interesting question, and we would expect it to depend on the problem at hand, as a core question is whether the features learned in the regression model learned from simulated data are of value also on data not stemming from the assumed model, but one subject to misspecification.

To some extent, we have touched this topic by considering outlier-corrupted data as a special case of model misspecification (Manuscript, Figure 6). We have shown in particular that the choice of robust norms such as L1 substantially mitigates the problem, see Section 3.4 in the manuscript.

While we unfortunately think that looking into this problem further would be beyond the scope of this paper, we have added the following clarification and remark to the discussion:

Changes in the manuscript:

Initial submission: -

Revised submission: While augmenting regression targets was essential to render inverse regression model based approaches applicable to non-identifiable forward models, one could further study the ramifications of the increased summary statistics dimension, as well as methods to automatically adjust the set of applied transformations. In particular, for multivariate problems it may be necessary to also consider combinations of parameters to render the inverse problem identifiable. While we showed that our approach also performed robustly in the presence of outliers for a given model, its applicability in general under model misspecification could be studied further [Frazier et al., 2020, Schmon et al., 2020].

Minor comments

1. I do not see where the notation S_{i_y,i_θ} has been defined. While it is fairly obvious what it is, it would be better to have it defined properly.

To improve the readability, we have changed the notation from $S_{i_y i_\theta}$ to S_{i_y,i_θ}. Further, we have added the following clarification:

Changes in the manuscript:

Initial submission: -

Revised submission: Here, S_{i_y,i_θ} denotes the sensitivity of the i_θth output with respect to the i_yth input.

2. The plot in Figure 4 is difficult to discern given the scale. Please correct this.

We agree that this plot is quite small. It is quite difficult to compress the large quantities of tabular information with a useful visual representation. We hope that the shown bars give a good overall indication, even if the single values cannot be all read easily. We have rotated the figure, which we hope is acceptable for the journal.

Changes in the manuscript:

Initial submission: Figure 2.

Revised submission: Figure 3.

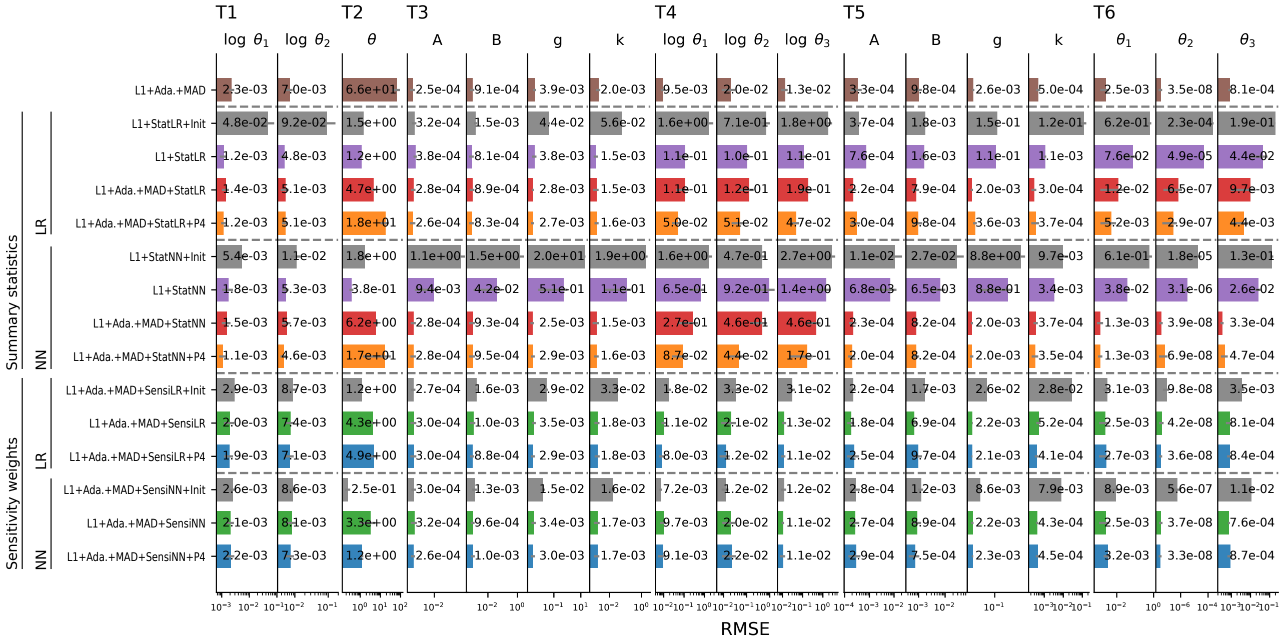

Figure 2: Median RMSE (smaller is better) for the parameters of models T1-6 (columns) obtained for 15 inference methods (rows), using an L1 distance, either uniformly weighted if unspecified, or with adaptive MAD scale normalization ("Ada.+MAD"). As regression models we considered linear regression ("LR") and neural networks ("NN"), both to define summary statistics ("Stat") and sensitivity weights ("Sensi"). Some inference settings further used parameter transformations $\lambda(\theta) = (\theta^1, \ldots, \theta^4)$ as regression targets ("P4"). In some settings, the regression model was trained before the initial generation ("Init"), or after 40% of the simulation budget if unspecified. The first row contains solely scale-normalized L1+Ada.+MAD as a reference, followed by two blocks of four rows using summary statistics, using firstly LR and secondly NN, and then by two blocks of three using sensitivity weights, using firstly LR and secondly NN. Reported values are medians over 10 replicates, with horizontal grey error lines indicating MAD.

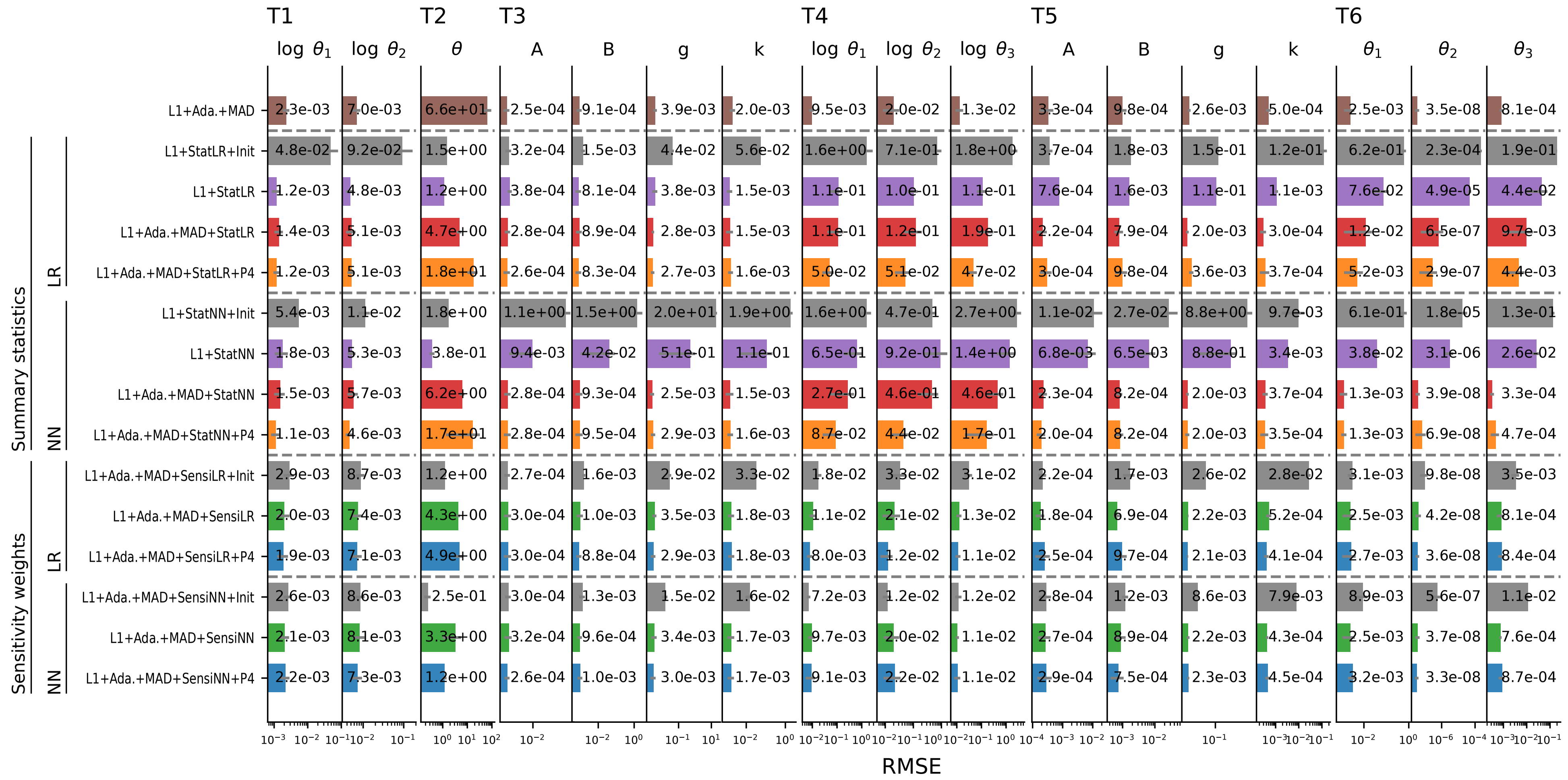

Figure 3: Median RMSE (smaller is better) for the parameters of models T1-6 (columns) obtained for 15 inference methods (rows), using an L1 distance, either uniformly weighted if unspecified, or with adaptive MAD scale normalization ("Ada.+MAD"). As regression models we considered linear regression ("LR") and neural networks ("NN"), both to define summary statistics ("Stat") and sensitivity weights ("Sensi"). Some inference settings further used parameter transformations $\lambda(\theta) = (\theta^1, \ldots, \theta^4)$ as regression targets ("P4"). In some settings, the regression model was trained before the initial generation ("Init"), or after 40% of the simulation budget if unspecified. The first row contains solely scale-normalized L1+Ada.+MAD as a reference, followed by two blocks of four rows using summary statistics, using firstly LR and secondly NN, and then by two blocks of three using sensitivity weights, using firstly LR and secondly NN. Reported values are medians over 10 replicates, with horizontal grey error lines indicating MAD.

Response to Reviewer 2

Summary: In this paper the authors present a method to adaptively reweight terms in the distance metric used in approximate Bayesian computation. These reweighting approach exploits sequential Monte Carlo (SMC) for ABC byt using early iterations to learn the inverse mapping from the data or summary statistics to parameters. For the training steps they consider both statistical (i.e., linera regression) and machine learing (i.e., neural networks). The key elements of there method is demonstrated on a specially constructed toy problem and then tested on a raneg of common benchmark models for ABC methods. Consistently their new approach is shown to have greater statistical efficiency and, more importantly, better robustness properties in the situation where data is corrupted by outliers.
I thoroughly enjoyed reading this paper as it addresses a very important problem for approximate Bayesian computation, that is the choice of distance metric and summary statistics. It was also refreshing to the see the problem addressed for the cases when the inverse mapping does not exist due to idendifiability concerns. While the statistically efficiency improvements are not always substantial, the robustness properties of this approach alone with its applicability to non-identifiable models make this a very useful piece of work. I therefore recommend it for publication pending some minor revisions based on my comments below.

We thank the reviewer for their assessment and can confirm that the manuscript message was accurately understood.

1. Regarding the main contribution (Algorithm 2), I have a few minor comments: 1.1 What tuning is involved in selecting the value for t_train? In the paper it seems that the only choices considered are t_train = 1 and t_train such that 40% of computational budget is used. How was the 40% budget chosen and what other considerations are important for choosing t_train?

We thank the reviewer for this comment, which we think we have not documented properly in the manuscript. It is correct that we consider either $t_{\text{train}} = 1$ or 40% of the simulation budget. The 40% was chosen heuristically based on a small number of tests.

We considered taking different values for the training time into account in our study, however this would have led to computational requirements we could not justify. We believe that further studies, beyond the scope of this work, can greatly improve upon this.

We have extended the discussion as follows:

Changes in the manuscript:

Initial submission: While in many cases delaying regression model training to later generations and a high-density region was advantageous, for simple models we observed benefits of early regression. Criteria on if and when to train or update regression models, also repeatedly, would be of interest.

Revised submission: While in many cases delaying regression model training to later generations and a high-density region was advantageous, for simple models we observed benefits of early regression. Besides training prior to the first generation, we only considered training the regression model after 40% of the simulation budget, based on the heuristic that the ABC-SMC algorithm should have had time to converge to a higher posterior density region, while still leaving a substantial simulation budget to leverage the learned mapping. However, in practice good training times will clearly be problem-specific. Criteria on if and when to train or update regression models, also repeatedly, would be of interest.

1.2 Throughout only L1 is used based on it's robustness properties. It would be interesting to see an examples of this adaptive scheme under L2 or another Lp in the presence of outliers. Given the discussed robustness properties of the adaptive reweighting, presumably this would better highlight this property compared to other approaches with L2 or other Lp distances.

We thank the reviewer for this comment. Indeed, we already did this in Schälte et al. [2021], where adaptive scale-normalized distances with L2 and L1 norm are contrasted (based on the paper by Prangle [2017]), and it is shown that the latter substantially increases robustness. We have no reason to believe that the method in the present manuscript, which adds informativeness assessment to the adaptive weighting, would perform any better on outlier-corrupted data with an L2 norm, as the regression model is based on simulated data and not designed to tackle model misspecification, and the ABC-SMC algorithm may have already concentrated on a "wrong" region in parameter space prior to regression model training.

Thus, we hope that Schälte et al. [2021] already answers this question sufficiently.

To point to further information on the topic of outliers, we added the following to the manuscript:

Changes in the manuscript:

Initial submission: -

Revised submission: For an in-depth study of adaptive scale normalization in the presence of data outliers, see Schälte et al. [2021].

1.3 I believe there is a typo in the second line of Algorithm 2 (for t = t_train, ..., n_t do), as the conditions in the subsquent if conditions (i.e., "if t < t_train then" and "if t+1 == t_trian") will alwasy be false.

We thank the reviewer for this observation. Indeed, it should read "from 1 to n_t" in the second line, as this loop describes the entire ABC-SMC workflow. We fixed this in the manuscript.

2. In section 2.2.3 when using multiple parameter moments to account for identifiability I am not completely clear on the implementation. Are you a) fitting k summaries independently (i.e., s_i from $y \sim \theta^i$) or b) using all the moments to obtain a single summary (i.e., s from $y \sim \theta + \theta^1 + ... + \theta^k$).

Indeed both implementations would be conceivable. Here, we fitted a single model from y to $\lambda(\theta) = (\theta^1, \dots, \theta^k) \in \mathbb{R}^{n_\theta \times k}$, i.e. the latter formulation in your comment. We believe this may leverage common structure better.

Changes in the manuscript:

Initial submission: When employing parameter augmentation (Section 2.2.3), we used the first four moments, $\lambda(\theta) = (\theta^1, \dots, \theta^4)$ ("P4"). We considered both regression to define summary statistics ("Stat", Section 2.2.1) and sensitivity weights ("Sensi", Section 2.2.2).

Revised submission: When employing parameter augmentation (Section 2.2.3), we used the first four moments, $\lambda(\theta) = (\theta^1, \dots, \theta^4)$ ("P4"). We considered both regression to define summary statistics ("Stat", Section 2.2.1) and sensitivity weights ("Sensi", Section 2.2.2). We used a single regression model to learn the full vector-valued mapping $y \mapsto \lambda(\theta)$, allowing to leverage synergies, as opposed to learning a separate model per regression target.

3. For the toy example, it is not completely clear how the synthetic dataset is constructed. Is each observsation a) a 5-d vector (with each component from the respective distibution), or b) a scalar randomly chosen from one of the components. If the answer is b) is each component equally likely, or are some rare events?

It is the former, however some of the data types are vector-valued themselves, such that we end up with at 17-dim vector, not 5-dim. We have clarified the introduction of the toy problem as follows:

Changes in the manuscript:

Initial submission: To illustrate the different problems addressed in this work, we constructed a demonstration problem with four parameters and five types of data:

Revised submission: To illustrate the different problems addressed in this work, we constructed a demonstration problem with four parameters and five types of data, constituting a joint data vector $y = (y_1, \ldots, y_5) \in \mathbb{R}^{17}$:

4. The following questions relate to the results: 4.1 In the results table in Figure 4, the vanilla L1+Ada.+MAD works surprisingly well in some cases. Can you provide some insight into this? I also presume that these results are for equivalent computational budgets.

We agree that the good performance of L1+Ada.+MAD on T3-6, where it was beaten only by few methods, is striking. All methods, including L1+Ada.+MAD, were given the same computational budget.

L1+Ada.+MAD only employs scale normalization, but no informativeness assessment. This would appear to indicate that on those problems, the data y are already sufficiently informative, in particular not confounded by uninformative statistics, such that an adaptive scale-normalization scheme suffices to guide the analysis. This in particular holds also on the high-dimensional ($n_y > 100$) problems T5+6.

To discuss this, we have extended the discussion of scale normalization in Section 3.3:

Changes in the manuscript:

Initial submission: -

Revised submission: It should further be remarked that overall, L1+Ada.+MAD performed quite robustly across all problems, including high-dimensional problems T5+6, and was consistently beaten by the methods accounting for informativeness only on T2. This again highlights the importance of scale normalization, and suggests that on these problems there were no overall uninformative data points (except T2) confounding the analysis.

4.2 For the tumor problem under the corruption of outliers why are only L1+Ada.+PCMAD+StatLR+P4 and L1+Ada.+PCMAD+SensiLR+P4 applied?

The tumor problem implementation we considered is, albeit written in C++, computationally highly expensive, with ABC-SMC runtimes on the order of hours even using hundreds of CPU cores. Thus, we had to subselect a few exemplary methods here and could not investigate the full range of methods.

To clarify this, we have reformulated in the main manuscript, Section 3.4:

Changes in the manuscript:

Initial submission: Given its computational complexity, we considered on this problem only selected approaches.

Revised submission: Given its computational complexity with ABC-SMC wall times on the order of several hours even with parallelization on hundreds of CPU cores, we had to restrict our analysis of this problem to only a few selected approaches.

5. There are a few discussion points that could be worth considering: 5.1. How do you deal with the case when rare "outlier" events are part of the data generating process.

Rare outliers (in the sense of e.g. Fujisawa et al. [2021]) can certainly confound any analysis. The treatment of rare outliers in the sense that only a few data points deviate from what the model can describe (e.g. experiment device failure, wrong labelling) is discussed in more depth in Schälte et al. [2021], which is at its core a simple study about L1 vs. L2 distances in combination with adaptive scale normalization. In Section 3.4, we show that our approach can deal with such scenarios.

A further question that may arise is what happens when the outliers are also modelled as part of the generative forward model, e.g. accounting for occasional device malfunction in the model $\pi(y|\theta)$. In that case, arguably theoretically our approach should still be able to recover all posterior expectations of interest, as with any other model. However, practically rare events arguably constitute a major problem to learning a meaningful regression model, at least requiring the use of large data sets for model training.

To address this question, and partly in response to another review comment on the curse of dimensionality, we have extended our discussion of the impact of forward model complexity on our approach:

Changes in the manuscript:

Initial submission: While simple linear models often sufficed, especially when trained on a high-density region, in some cases more complex models were superior. A systematic investigation of alternative and more complex model types, e.g. neural networks tailored to the respective data types, as well as model selection, would be useful. While increasing the training set, which may be beneficial especially for complex models and lead to more robust estimators, is straightforward, as it only requires continued sampling from the forward model, there is a cost trade-off of the actual ABC inference and regression model training.

Revised submission: While simple linear models often sufficed, especially when trained on a high-density region, in some cases more complex models were superior, indicating the presence of non-linear data-parameter relationships. A systematic investigation of alternative and more complex regression model types, e.g. neural networks tailored to the respective data types, as well as model selection among competing regression models, would be useful. To select among competing model candidates, an efficient yet robust model selection scheme with out-of-sample evaluation, e.g. based on cross validation, might be useful. The employed regression model should reflect and exploit any symmetries or structures in the data. For example, for image and sequence data convolutional and recurrent networks would be prime candidates, for i.i.d. data permutation invariant networks [Radev et al., 2020]. More complex regression models and robust estimators require larger training sets (which could be mitigated by transfer learning). While increasing the training set is straightforward, as it only requires continued sampling from the forward model, there is a cost trade-off of the actual ABC inference and regression model training, which remains to be investigated.

5.2. beyond corruption of outliers, there are other kinds of miss-specification that are structural (e.g., using an SIR model when the real process is SEIR). Could your approach be used to identify this?

Unfortunately, our approach would most certainly not be able to correct for miss-specifications in such scenarios. The approach is based on the assumption that the "bulk" of the data can be fitted by the model and that only relatively few data points can apparently not be explained by the model, in which case we can label them as potential outliers in a simulation-based manner. However, our approach could conceptually be integrated in a model selection framework, which could allow to decide e.g. SIR vs. SEIR by inspection of Bayes factors (see also a response to Reviewer 3 on model selection).

5.3 I believe this adaptive scheme could be particularly valuable for multifidelity method which are methods that exploit approximate models and appropriately correct for bias. Could your method be applied to automatically construct summaries/distances for each model fidelity, such that the ROC properties of the approximations are improved (and therefore less need to simulated the exact model). This could be particularly useful within Adaptive mutlifidelity schemes such as
https://doi.org/10.1137/20M1316160
https://arxiv.org/abs/2112.11971
https://doi.org/10.1016/j.jcp.2022.111543

Multifidelity approaches are certainly highly interesting in that they hold the potential to substantially reduce the required number of expensive model evaluations. To the best of our knowledge, adaptive distance schemes have not yet been considered in a multifidelity scheme, but the distance metrics have been pre-determined (e.g. as an L2 distance). However, firstly adaptive thresholding schemes such as Drovandi and Pettitt [2011], Silk et al. [2013], and secondly adaptive distances such as Prangle [2017], or of course the present work, should be straightforward to integrate into multifidelity schemes, which make no assumption about the nature of the acceptance decision, given some regularity conditions hold.

To discuss this point, we have extended the discussion:

Changes in the manuscript:

Initial submission: -

Revised submission: We have shown here that adaptive distance metrics accounting for both scale and informativeness facilitate an efficient analysis. We envisage that they may prove valuable also in combination with many orthogonal methods in an ABC-SMC framework. Promising appear for example a combination with optimal thresholding schemes [Silk et al., 2013], as well as a combination with multifidelity approaches using surrogates to even further reduce the number of evaluations of expensive simulators [Prescott and Baker, 2021].

6. Some minor typographical things throughout that I found, suggest a careful proof-read:
6.1 Line 30 "allowing to understand"
6.2 Line 33 "allows doing so"
6.3 Line 37 "In a nutshell" is a bit informal suggest "Put briefly"
6.4 Line 39 "This way," → "In this way,"
6.5 Line 44 "demonstrate" → "demonstrates"
6.6 Lines 54-55 sentence is a bit awkward, suggest rephrasing.
6.7 throughout section 2: define all maths symbols e.g., n_y n_s and n_theta are never defined
6.8 Line 105 "Monte-Carlo" → "Monte Carlo"
6.9 Line 214 define PCMAD

We thank the reviewer for these observations, have incorporated all changes accordingly and proof-read the manuscript.

In particular, we addressed 6.7 as:

Changes in the manuscript:

Initial submission: -

Revised submission: Here and throughout this manuscript, n_{var} denotes the dimension of a given variable var.

6.9 we addressed as:

Changes in the manuscript:

Initial submission: To further reduce the impact of outliers, we complement MAD, as a measure of sample variability, by the median absolute deviation to the observed value, as a measure of deviation, giving a normalization term PCMAD (see Schälte et al. [2021] for details).

Revised submission: To further reduce the impact of outliers, we complement MAD, as a measure of sample variability, by the median absolute deviation to the observed value, as a measure of deviation, giving a normalization term PCMAD ("perhaps use the combined median absolute deviation (CMAD) to the population median (MAD) and to the observation (MADO), or only use MAD"; see Schälte et al. [2021] for details).

Response to Reviewer 3

Using NN to improve summary statistics of improve ABC is a nice idea. I posed three questions in my review of the manuscirpt concerning non-identifiability, curse of dimensionality and conditions for model selection.
The authors introduce regression-based summary statistics to over- come three problems that may arise in ABC: 1. From partly uninformative data for scale-normalized distances 2. From heterogeneous parameter scales for regression-based summary statistics 3. From parameter non-identifiability for regression model adequacy The manuscript contributions are partly based on NN models. This is an important application which has received broad attention recently [1]. The manuscript's findings are of interest to the readers of PLOS ONE, and I recommend proceeding with the manuscript publication after a few issues listed below are addressed.

We thank the reviewer for their assessment and can confirm that the manuscript was correctly understood. We provide a response to each of the three questions below.

Model selection Does Theorem 1 in the supplementary information imply conditions for model selection, e.g., in the sense of Marin et al. [3]? You mention model selection as part of the future work in the discussion of the manuscript. Model selection would make pyABC powerful.

The tool pyABC already supports model selection via a hierarchical joint-space approach from Toni and Stumpf [2010], see e.g. `https://pyabc.readthedocs.io/en/latest/examples/model_selection.html`. Yet, a core problem with model selection in ABC is that summary statistics $s(y)$ can lead to inconsistent Bayes factors. Given models M_1 and M_2, only if the condition $\pi(y_{\text{obs}}|s(y_{\text{obs}}), M_1) = \pi(y_{\text{obs}}|s(y_{\text{obs}}), M_2)$ is satisfied, will calculated Bayes factors be consistent with those based on the full data. Sufficiency for the individual models is in general not sufficient to decide the model selection problem [Didelot et al., 2011].

We are presently not aware of how Theorem 1 in the Supplementary Information, which proves convergence of posterior moments under suitable corresponding summary statistics would translate to a model selection scenario (but we would be interested in further discussion on this topic). However, what we have shown is that the adaptive weighting scheme accounting for scale and informativeness allows to circumvent the use of summary statistics altogether, similar to what is proposed in Bernton et al. [2019] via Wasserstein distances. In this case, calculated Bayes Factors will be asymptotically consistent with the true Bayes factors.

When we refer to model selection in the discussion, we imply selection among suitable regression models. We clarified this as follows:

Changes in the manuscript:

Initial submission: A systematic investigation of alternative and more complex model types, e.g. neural networks tailored to the respective data types, as well as model selection, would be useful.

Revised submission: A systematic investigation of alternative and more complex model types, e.g. neural networks tailored to the respective data types, as well as model selection among competing regression models, would be useful. To select among competing model candidates, an efficient yet robust model selection scheme with out-of-sample evaluation, e.g. based on cross validation, might be useful.

In order to discuss possible implications for model selection, we further added the following to the discussion:

Changes in the manuscript:

Initial submission: -

Revised submission: A core problem with model selection in ABC is that insufficient summary statistics can lead to inconsistent Bayes factors [Didelot et al., 2011]. Thus, approaches such as our adaptive weights accounting for scale and informativeness, or also Harrison and Baker [2020] and Bernton et al. [2019], hold the potential to facilitate unbiased ABC model selection even on larger data sets.

Curse of dimensionality The results in the manuscript are applied to a somewhat large number of low-dimensional examples. I miss some candid insight on where the limitations are while using the manuscript results in high dimensional parameter space. How would the NN model need to be adapted? ("...NN models were considered with a single hidden layer of dimension $[(n_y + n_\theta)/2]$, with ReLU activation function, using ADAM stochastic gradient descent for optimization, and early stopping to avoid overfitting, with a 10% validation set....").

Indeed the largest model we considered in this study was the 7-parameters agent-based tumor growth model, which for us already constituted a challenging application, mainly due to its computational complexity and some interesting parameter properties. In our field, we have scarcely seen ABC applied to models beyond, say, 10 parameters, however in other research areas this is of course different. Thus, a further study on more complex, higher-dimensional problems may be of interest.

We would argue that per se the NN complexity does not need to increase with increased parameter or data dimension, as it depends on the complexity of the mapping $y \mapsto \theta$. We adjusted the hidden dimension in linear dependence on n_y, n_θ to reflect the increase in dimension, however did not investigate how e.g. and increased depth would be beneficial. In contrast, in Jiang et al. [2017] a DNN with 3 hidden layers of (fixed) dimension 100 is used. While a deeper/larger NN model increases expressiveness, it comes at the drawback of requiring more samples to train (e.g. 1e6 in Jiang et al. [2017].

What we believe to be of substantial value is that the employed regression/NN model should reflect and exploit any symmetries or structures in the data. For example, for image and sequence data convolutional and recurrent networks would be prime candidates, for i.i.d. data permutation invariant networks, see Radev et al. [2020] for a discussion in a related context.

To clarify what we believe to hold in these cases, we have reformulated and extended the corresponding paragraph:

Changes in the manuscript:

Initial submission: While simple linear models often sufficed, especially when trained on a high-density region, in some cases more complex models were superior. A systematic investigation of alternative and more complex model types, e.g. neural networks tailored to the respective data types, as well as model selection, would be useful. While increasing the training set, which may be beneficial especially for complex models and lead to more robust estimators, is straightforward, as it only requires continued sampling from the forward model, there is a cost trade-off of the actual ABC inference and regression model training.

Revised submission: While simple linear models often sufficed, especially when trained on a high-density region, in some cases more complex models were superior , indicating the presence of non-linear data-parameter relationships. A systematic investigation of alternative and more complex regression model types, e.g. neural networks tailored to the respective data types, as well as model selection among competing regression models, would be useful. To select among competing model candidates, an efficient yet robust model selection scheme with out-of-sample evaluation, e.g. based on cross validation, might be useful. The employed regression model should reflect and exploit any symmetries or structures in the data. For example, for image and sequence data convolutional and recurrent networks would be prime candidates, for i.i.d. data permutation invariant networks [Radev et al., 2020]. More complex regression models and robust estimators require larger training sets (which could be mitigated by transfer learning). While increasing the training set is straightforward, as it only requires continued sampling from the forward model, there is a cost trade-off of the actual ABC inference and regression model training, which remains to be investigated.

Non-identifiability I fail to understand the non-identifiability remedy presented in the results. Wouldn't it make sense to say a model is identifiable is the parameter to observable mapping (in the zero noise limit) is injective, e.g., in the sense of Bellu et al. [2], and have a separate name for the result shown in the manuscript? If we a have population growth model with both intrinsic growth rate and carrying capacity, but the corresponding data only informs the growth regime, then the carrying capacity is non-identifiable. Is it possible to apply the manuscript findings to make the carrying capacity identifiable?

We thank the reviewer for this comment, which is also in line with a comment by Reviewer 1. To clarify the topic, we implemented several changes (also in response to other comments). Indeed, the problem we are addressing is that established approaches based on Fearnhead and Prangle [2012] cannot handle the situation that some parameters cannot be expressed as functions of the data, even in the zero-noise limit.

In a logistic growth model, i.e. a population growth model with limiting capacity, if the carrying capacity is not identifiable from the data, e.g. because the data only cover the growth regime, then our method will not be able to render it identifiable. From how we understand it, there cannot be any inference method rendering the parameter identifiable, simply because there are no data to make accurate inference about it. This would require an extension of the data set, or may even require new experiments to observe latent species. However, if e.g. only a combination of parameters is identifiable, but not the individual parameters, then the approach Fearnhead and Prangle [2012] may fail to construct a meaningful inverse regression model, while our approach of augmenting the regression targets may work superior. This means that it may uncover e.g. a bimodality in the parameters, while the original approach by Fearnhead and Prangle [2012] may detect no information about the parameters at all.

To clarify this point as well as points of other reviewers, we implemented various changes in the manuscript:

Changes in the manuscript:

Initial submission: A further problem with regression-based statistics is that an inverse mapping may not always exist, e.g. when parameters are not globally identifiable.

Revised submission: A further problem with regression-based statistics is that a unique inverse mapping from data to parameters may not always exist even in the noise-free limit, resulting e.g. in a multi-modal posterior distribution, or plateaus of combinations of parameter values achieving the same posterior value. This is in particular the case when the observable model outputs and data are not rich enough to allow to structurally identify the parameters globally [Wieland et al., 2021].

Changes in the manuscript:

Initial submission: A problem with inverse regression models of parameters on data is that such a mapping may not exist. For example, consider a quadratic model $y \sim \mathcal{N}(\theta^2, 0.1^2)$, with prior $\theta \sim U[-1, 1]$, and observed data $y_{\text{obs}} = 0.7$. [...]
A solution is to consider transformations $\lambda(\theta)$ of the parameters [...]

Revised submission: A problem with inverse regression models of parameters on data is that such a mapping may not exist. For example, consider a quadratic model $y \sim \mathcal{N}(\theta^2, 0.1^2)$, with prior $\theta \sim U[-1, 1]$, and observed data $y_{\text{obs}} = 0.7$. Given the data, the underlying parameter cannot be uniquely identified. [...]
In general, non-identifiability can be tackled in various ways, e.g. by fixing some parameters to constant values, reparameterization, or by generating additional data to break the symmetry [Wieland et al., 2021]. However, this requires an in-depth and iterative model analysis. Even for deterministic models based on ordinary differential equation, this is already a challenging task [Villaverde et al., 2021], while ABC is commonly applied to highly complex stochastic models. Further, one ends up with an altered inference problem, which renders the analysis of prediction uncertainties more involved [Villaverde et al., 2021].
Here we propose an approach that is able to work with the original, unaltered, problem formulation, including potential non-identifiabilities. We solve the problem by considering transformations $\lambda(\theta)$ of the parameters. [...]

Changes in the manuscript:

Initial submission: -

Revised submission: While augmenting regression targets was essential to render inverse regression model based approaches applicable to non-identifiable forward models, one could further study the ramifications of the increased summary statistics dimension, as well as methods to automatically adjust the set of applied transformations. In particular, for multivariate problems it may be necessary to also consider combinations of parameters to render the inverse problem identifiable. While we showed that our approach also performed robustly in the presence of outliers for a given model, its applicability in general under model misspecification could be studied further [Frazier et al., 2020, Schmon et al., 2020].

References

Bernton, E. et al. Approximate bayesian computation with the wasserstein distance. J. Roy. Stat. Soc. B (Statistical Methodology), 81(2):235–269, 2019.

Blum, M.G. et al. A comparative review of dimension reduction methods in approximate Bayesian computation. Statistical Science, 28(2):189–208, 2013.

Didelot, X. et al. Likelihood-free estimation of model evidence. Bayesian analysis, 6(1):49–76, 2011.

Drovandi, C.C. and Pettitt, A.N. Estimation of parameters for macroparasite population evolution using approximate Bayesian computation. Biometrics, 67(1):225–233, 2011.

Fearnhead, P. and Prangle, D. Constructing summary statistics for approximate Bayesian computation: semi-automatic approximate Bayesian computation. J. R. Stat. Soc. B, 74(3): 419–474, 2012.

Frazier, D.T. et al. Asymptotic properties of approximate bayesian computation. Biometrika, 105(3):593–607, 2018.

Frazier, D.T. et al. Model misspecification in approximate Bayesian computation: consequences and diagnostics. J. R. Stat. Soc. B, 2020.

Fröhlich, F. et al. Multi-experiment nonlinear mixed effect modeling of single-cell translation kinetics after transfection. npj Systems Biology and Applications, 5(1):1, 2018. doi: 10.1038/s41540-018-0079-7. URL `https://doi.org/10.1038/s41540-018-0079-7`.

Fujisawa, M. et al. γ-ABC: Outlier-robust approximate Bayesian computation based on a robust divergence estimator. In International Conference on Artificial Intelligence and Statistics, pages 1783–1791. PMLR, 2021.

Harrison, J.U. and Baker, R.E. An automatic adaptive method to combine summary statistics in approximate bayesian computation. PloS one, 15(8):e0236954, 2020.

Jiang, B. et al. Learning summary statistic for approximate Bayesian computation via deep neural network. Statistica Sinica, pages 1595–1618, 2017.

Li, W. and Fearnhead, P. On the asymptotic efficiency of approximate bayesian computation estimators. Biometrika, 105(2):285–299, 2018.

Prangle, D. Adapting the ABC distance function. Bayesian Analysis, 12(1):289–309, 2017.

Prescott, T.P. and Baker, R.E. Multifidelity approximate Bayesian computation with sequential Monte Carlo parameter sampling. SIAM/ASA Journal on Uncertainty Quantification, 9(2): 788–817, 2021.

Radev, S.T. et al. Bayesflow: Learning complex stochastic models with invertible neural networks. IEEE transactions on neural networks and learning systems, 2020.

Schälte, Y. et al. Robust adaptive distance functions for approximate Bayesian inference on outlier-corrupted data. bioRxiv, 2021.

Schmon, S.M. et al. Generalized posteriors in approximate bayesian computation. arXiv preprint arXiv:2011.08644, 2020.

Silk, D. et al. Optimizing threshold-schedules for sequential approximate Bayesian computation: Applications to molecular systems. Stat. Appl. Genet. Mol. Biol., 12(5):603–618, Oct. 2013.

Toni, T. and Stumpf, M.P.H. Simulation-based model selection for dynamical systems in systems and population biology. Bioinformatics, 26(1):104–110, 10 2010.

Villaverde, A.F. et al. A protocol for dynamic model calibration. Briefings in Bioinformatics, 10 2021. ISSN 1477-4054. doi: 10.1093/bib/bbab387. URL `https://doi.org/10.1093/bib/bbab387`. bbab387.

Wieland, F.G. et al. On structural and practical identifiability. Current Opinion in Systems Biology, 25:60–69, 2021.
