# Supplementary material for: Informative and adaptive distances and summary statistics in sequential approximate Bayesian computation
Source: PLoS One. 2023 May 22;18(5):e0285836. doi: 10.1371/journal.pone.0285836 (PMC10202307; doi:10.1371/journal.pone.0285836)
Supplement: Attachment — Submitted filename: JPONE_D-22-20368_report_R1.pdf [file pone.0285836.s005.pdf]

PONE-D-22-20368: R1

February 28, 2023

1 Summary

The authors have now responded to my comments, and their revisions have significantly improved the paper. I have a few remaining minor comments I would like the authors to look at before finalizing the paper.

2 General Comments

Previous Comment (1): Differentiability Issues. In regards to my previous comments about the differentiability issues, I was referring to the differentiability of the summaries specifically in the case of finite-samples. That is, my comment was specifically about the sensitivity matrix defined in (3), which is a derivative, wrt the data y, of the summary mapping $s : \mathbb{R}^{d_y} \to \mathbb{R}^{d_\theta}$. In the case of sample quantiles, yes, this map is differentiable in expectation, but the resulting map $s(y)$ is not differentiable for any finite-sample. Moreover, employing such an estimator will ultimately deliver a biased estimator for the derivative you wish to approximate.

The authors have not adequately addressed this issue in the text, as far as I can see, and their response to my previous comment does not solve the issue above. This lack of exchange between differentiation and integration in finite-samples should be acknowledged as a limitation of the method.

Previous comment (5): Inverse Mapping Frazier et al. (2018) and Li and Fearnhead (2018) do not use the nomenclature 'inverse mapping' due to the fact that the standard name for the mapping $\theta \mapsto s(y)$, where $y \sim p(\cdot|\theta)$, is the (sample) binding function, which comes from a long line of research in simulated frequentist statistics (Indirect Inference more precisely). Nonetheless, this is precisely the inverse mapping that matters for the statistical analysis as it allows you to recover/identify θ by 'inverting' the limit version of this map. The non-identifiablity, i.e., the lack of a unique inverse mapping in the authors nomenclature, appears to be the culprit behind this particular issue with the combination of model and regression summaries. In that way, I am wondering if this is a break-down of the identification conditions employed in the theoretical study of ABC and other simulated methods, such as Assumption 3(ii) in Frazier et al. (2018), and 4(ii) in Li and Fearnhead (2018). Please clarify in the text this issue.
