# Supplementary material for: Informative and adaptive distances and summary statistics in sequential approximate Bayesian computation
Source: PLoS One. 2023 May 22;18(5):e0285836. doi: 10.1371/journal.pone.0285836 (PMC10202307; doi:10.1371/journal.pone.0285836)
Supplement: Attachment — Submitted filename: Response2_Informative.pdf [file pone.0285836.s006.pdf]

Author's response to editor's and reviewers' comments

For the paper: "*Informative and adaptive distances and summary statistics in sequential approximate Bayesian computation*".

We thank the editor and the reviewers for their comments. Based on their remarks, we have revised the manuscript and the supplementary information. We addressed all their comments and resolved the raised issues. Our point-by-point response is provided below.

The teal boxes contain the editor's and the reviewers' comments, while the yellow boxes contain the changes made in the manuscript. To provide some context, changes (blue) are sometimes accompanied by unchanged text (black).

Response to Reviewer 1

1 Summary
The authors have now responded to my comments, and their revisions have significantly improved the paper. I have a few remaining minor comments I would like the authors to look at before finalizing the paper.

We thank the reviewer for their previous comments and their assessment. We provide a response to the remaining minor comments below.

2 General Comments
Previous Comment (1): Differentiability Issues. In regards to my previous comments about the differentiability issues, I was referring to the differentiability of the summaries specifically in the case of finite-samples. That is, my comment was specifically about the sensitivity matrix defined in (3), which is a derivative, wrt the data y, of the summary mapping $s : \mathbb{R}^{d_y} \to \mathbb{R}^{d_\theta}$. In the case of sample quantiles, yes, this map is differentiable in expectation, but the resulting map $s(y)$ is not differentiable for any finite-sample. Moreover, employing such an estimator will ultimately deliver a biased estimator for the derivative you wish to approximate. The authors have not adequately addressed this issue in the text, as far as I can see, and their response to my previous comment does not solve the issue above. This lack of exchange between differentiation and integration in finite-samples should be acknowledged as a limitation of the method.

We thank the reviewer for this clarification. We have extended the discussion to acknowledge this limitation of the method:

Changes in the manuscript:

Initial submission: It should be noted that in calculating sensitivity weights we make the assumption that the employed regression model is (sub-)differentiable, which clearly holds for the regression models employed in this work (see also the Supplementary Information, Section 3). Further, we implicitly assume that the employed smooth regression model provides an accurate approximation of the underlying expectations it aims to describe. In practice, it should be carefully evaluated whether such an approximation appears valid for the given forward model.

Revised submission: It should be noted that in calculating sensitivity weights we make the assumption that the employed regression model is (sub-)differentiable, which clearly holds for the regression models employed in this work (see also the Supplementary Information, Section 3). Further, we implicitly assume that the employed smooth regression model provides an accurate approximation of the underlying expectations it aims to describe. In practice, it should be carefully evaluated whether such an approximation appears valid for the given forward model. In the first place, we use the derived sensitivity matrix as a "heuristic" to quantify informativeness for weighting. However, it may fail in this function as a heuristic if the underlying quantities are inadequately captured. In particular, it provides a biased approximation of the derivative it intends to approximate when in the underlying expectations integration and differentiation cannot be exchanged in finite samples. Studying the consequences of such conceptual and practical discrepancies as well as investigating alternative measures of sensitivity could improve robustness and applicability of the method.

Previous comment (5): Inverse Mapping. Frazier et al. (2018) and Li and Fearnhead (2018) do not use the nomenclature 'inverse mapping' due to the fact that the standard name for the mapping $\theta \mapsto s(y)$, where $y \sim p(\cdot|\theta)$, is the (sample) binding function, which comes from a long line of research in simulated frequentist statistics (Indirect Inference more precisely). Nonetheless, this is precisely the inverse mapping that matters for the statistical analysis as it allows you to recover/identify θ by 'inverting' the limit version of this map. The non-identifiablty, i.e., the lack of a unique inverse mapping in the authors nomenclature, appears to be the culprit behind this particular issue with the combination of model and regression summaries. In that way, I am wondering if this is a break-down of the identification conditions employed in the theoretical study of ABC and other simulated methods, such as Assumption 3(ii) in Frazier et al. (2018), and 4(ii) in Li and Fearnhead (2018). Please clarify in the text this issue.

We thank the reviewer for this comment. To clarify our meaning of "inverse", we have added the following to the introduction:

Changes in the manuscript:

Initial submission: In a popular class of approaches, inverse regression models of parameters on simulated data have been used as statistics [Borowska et al., 2021, Fearnhead and Prangle, 2012, Jiang et al., 2017].

Revised submission: In a popular class of approaches, inverse regression models of parameters on simulated data have been used as statistics [Borowska et al., 2021, Fearnhead and Prangle, 2012, Jiang et al., 2017]. Here, by "inverse" we mean that the summary statistics map from (functions of) simulated data back to (functions of) the parameters, i.e. in the inverse direction to the forward mechanistic model.

Further, we have clarified the identifiability issue and put it into context of the mentioned theoretical work, by the following discussion in the methods section:

Changes in the manuscript:

Initial submission: -

Revised submission: More generally, an informative inverse mapping from data back to parameters can only exist when the forward model $\theta \mapsto \pi(\cdot|\theta)$ is injective, i.e. the model is structurally identifiable in the limit of infinite data [Lehmann and Casella, 2006, Raue et al., 2009]. We would like to remark that this lack of identifiability of true parameters given data also stands in the way of theoretical asymptotic results obtained for ABC methods regarding convergence of point estimators in the large-data limit in Li and Fearnhead [2018] (Condition 4.(ii)) and limiting shape of posteriors and posterior means in Frazier et al. [2018] (Assumption 3.(ii)).

Response to Reviewer 2

I am very pleased with the responses by the authors. All my questions and comments have been addressed.
I believe this will be a very useful paper to the systems biology community, and statistical computing more broadly.

We thank the reviewer for this assessment, as well as for their time and valuable comments.

Response to Reviewer 3

The authors have addressed all my concerns. I appreciate their careful and insightful replies. In particular, the one about model selection.

We thank the reviewer for this assessment, as well as for their time and valuable comments.

References

Borowska, A. et al. Gaussian process enhanced semi-automatic approximate Bayesian computation: parameter inference in a stochastic differential equation system for chemotaxis. Journal of Computational Physics, 429:109999, 2021.

Fearnhead, P. and Prangle, D. Constructing summary statistics for approximate Bayesian computation: semi-automatic approximate Bayesian computation. J. R. Stat. Soc. B, 74(3): 419–474, 2012.

Frazier, D.T. et al. Asymptotic properties of approximate bayesian computation. Biometrika, 105(3):593–607, 2018.

Jiang, B. et al. Learning summary statistic for approximate Bayesian computation via deep neural network. Statistica Sinica, pages 1595–1618, 2017.

Lehmann, E.L. and Casella, G. Theory of point estimation. Springer Science & Business Media, 2006.

Li, W. and Fearnhead, P. On the asymptotic efficiency of approximate bayesian computation estimators. Biometrika, 105(2):285–299, 2018.

Raue, A. et al. Structural and practical identifiability analysis of partially observed dynamical models by exploiting the profile likelihood. Bioinformatics, 25(25):1923–1929, May 2009. doi: 10.1093/bioinformatics/btp358.
